# Chronic Apocynin Treatment Attenuates Beta Amyloid Plaque Size and Microglial Number in hAPP(751)_SL_ Mice

**DOI:** 10.1371/journal.pone.0020153

**Published:** 2011-05-31

**Authors:** Melinda E. Lull, Shannon Levesque, Michael J. Surace, Michelle L. Block

**Affiliations:** Department of Anatomy and Neurobiology, Virginia Commonwealth University Medical Campus, Richmond, Virginia, United States of America; Nathan Kline Institute and New York University School of Medicine, United States of America

## Abstract

**Background:**

NADPH oxidase is implicated in neurotoxic microglial activation and the progressive nature of Alzheimer's Disease (AD). Here, we test the ability of two NADPH oxidase inhibitors, apocynin and dextromethorphan (DM), to reduce learning deficits and neuropathology in transgenic mice overexpressing human amyloid precursor protein with the Swedish and London mutations (hAPP(751)_SL_).

**Methods:**

Four month old hAPP(751)_SL_ mice were treated daily with saline, 15 mg/kg DM, 7.5 mg/kg DM, or 10 mg/kg apocynin by gavage for four months.

**Results:**

Only hAPP(751)_SL_ mice treated with apocynin showed reduced plaque size and a reduction in the number of cortical microglia, when compared to the saline treated group. Analysis of whole brain homogenates from all treatments tested (saline, DM, and apocynin) demonstrated low levels of TNFα, protein nitration, lipid peroxidation, and NADPH oxidase activation, indicating a low level of neuroinflammation and oxidative stress in hAPP(751)_SL_ mice at 8 months of age that was not significantly affected by any drug treatment. Despite *in vitro* analyses demonstrating that apocynin and DM ameliorate Aβ-induced extracellular superoxide production and neurotoxicity, both DM and apocynin failed to significantly affect learning and memory tasks or synaptic density in hAPP(751)_SL_ mice. To discern how apocynin was affecting plaque levels (plaque load) and microglial number *in vivo*, *in vitro* analysis of microglia was performed, revealing no apocynin effects on beta-amyloid (Aβ) phagocytosis, microglial proliferation, or microglial survival.

**Conclusions:**

Together, this study suggests that while hAPP(751)_SL_ mice show increases in microglial number and plaque load, they fail to exhibit elevated markers of neuroinflammation consistent with AD at 8 months of age, which may be a limitation of this animal model. Despite absence of clear neuroinflammation, apocynin was still able to reduce both plaque size and microglial number, suggesting that apocynin may have additional therapeutic effects independent of anti-inflammatory characteristics.

## Introduction

Alzheimer's disease (AD) is a devastating and progressive neurodegenerative disease that culminates in dementia, affecting over 5 million people in the United States alone. Current treatment is largely unable to halt disease progression. The hallmark neuropathology of AD consists of insoluble extracellular plaques containing β -amyloid (Aβ) and intraneuronal neurofibrillary tangles in the cortical region of the brain. Microglia, the resident immune cells in the brain, have been implicated in the progressive nature of numerous neurodegenerative diseases, particularly AD [1]. However, traditional anti-inflammatory therapies such as Non-steroidal Anti-inflammatory Drugs (NSAIDs) have produced conflicting results [2], highlighting the need for new and more specific anti-inflammatory targets. Here, we propose that targeting NADPH oxidase and neurotoxic microglial activation may be of significant therapeutic relevance for AD.

NADPH oxidase is an enzyme complex in phagocytes, such as microglia, that is activated during host defense to catalyze the production of superoxide from oxygen [3]. A variety of stimuli, including bacteria components [4], inflammatory peptides [3], Aβ [5], and multiple other neurotoxins [6] activate microglial NADPH oxidase, causing the production of neurotoxic reactive oxygen species (ROS). In fact, NADPH oxidase is activated in the brains of AD patients [7] and the catalytic subunit (gp91) is upregulated in Parkinson's disease (PD) [8], further implicating the enzyme complex in neurodegenerative diseases.

The premise of deleterious microglial activation in AD has been supported by analysis of post-mortem brains from AD patients [9], [10], where microglial activation occurred before neuropil damage in the disease process [11], suggesting a causal role. The Amyloid Hypothesis holds that Aβ has a causative role in AD pathology, which may occur through direct toxicity to neurons [12], [13] and microglia-mediated neurotoxicity [14], [15]. In fact, evidence shows that microglia cluster around senile plaques and neurofibrillary tangles [10], [16], become activated [17], and produce neurotoxic factors, including nitric oxide (NO) [18], superoxide [15], [19], and tumor necrosis factor alpha (TNFα) [20]. Several studies have demonstrated that Aβ will both recruit and activate microglia [16], [17], further supporting a role for both Aβ and microglia in AD progression [21]. Interestingly, the receptor complex necessary for microglia to recognize and phagocytize Aβ fibrils are also the same receptors responsible for Aβ activation of microglial NADPH oxidase and the production of superoxide [22], [23], indicating microglia themselves are a source of oxidative stress [23]. Furthermore, microglial NADPH oxidase has also been implicated as a critical component to neurotoxic reactive microgliosis [24], [25], [26]. Reactive microgliosis refers to the toxic microglial response to neuronal damage responsible for a chronic cycle of neuroinflammation and neurotoxicity [27], a process believed to underlie diverse neurodegenerative diseases [24], [25], [26].

Several compounds have demonstrated the ability to inhibit microglial NADPH oxidase, including memantine [28], [29], statins [30], ibuprophin [5], dextromethorphan (DM) [31], [32], [33], and 4′-Hydroxy-3′-methoxyacetophenone (apocynin) [34]. DM is a noncompetitive N-methyl-d aspartate (NMDA) receptor agonist that has been shown to have both anti-inflammatory [31] and neuroprotective properties in models of Parkinson's disease [31], [32], [33], independent of the NMDA receptor and through inhibition of microglial NADPH oxidase [35], [36]. Apocynin was originally isolated from the medicinal plant *Picrorhiza kurroa,* has low toxicity, impairs the assembly of the NADPH oxidase complex [37], and is neuroprotective against microglia-mediated neurotoxicity in PD models [38]. While clearly successful in PD models [31], [32], [33], until now the ability of DM and apocynin to offer *in vivo* neuroprotection in AD models was unknown.

In the current study, we addressed whether chronic administration of known NADPH oxidase inhibitors (apocynin and DM), beginning at the time plaque deposition began to occur in hAPP(751)_SL_ mice, could prevent neuroinflammation, neuron damage, and behavioral learning and memory deficits.

## Materials and Methods

### Reagents

Lipopolysaccharide (LPS; strain O111:B4) was purchased from EMD Chemicals (Gibbstown, NJ). Cell culture reagents were obtained from Invitrogen (Carlsbad, CA). HALT protease inhibitor was obtained from Thermo Fisher Scientific (Rockford, IL). Fluorescent Aβ peptide was purchased from AnaSpec, Inc. (Fremont, CA), and non-fluorescent Aβ was purchased from American Peptide Company (Sunnyvale, CA). Dextromethorphan, apocynin, staurosporine, fMetLeuPhe and all other reagents were purchased from Sigma Aldrich Chemical Co. (St. Louis, MO).

### Animals

A total of 53 male transgenic hAPP(751)_SL_ mice with a C57BL/6xCBA background were used for the *in vivo* portion of this study. The hAPP(751)_SL_ mice over-express human APP(751) with the London (V717I) and the Swedish (K670M/N671L) mutations under the regulatory control of the murine-Thy-1 promoter, which ensures high expression in brain neurons, with little expression in the periphery. Due to the London mutation, high levels of β-amyloid 1–42 are expressed all over the brain, particularly in the cortex and hippocampus. The hAPP(751)_SL_ mice develop plaques consisting of amyloid depositions starting at 3 to 4 months, where deposits begin to accumulate in the hippocampus by 7 months. The hAPP(751)_SL_ mice also show neuronal damage with increasing age, particularly at 14 months [39]. The hAPP(751)_SL_ mice fail to show motor deficits, but present profound deficits for tests of cognition, including the Morris Water Maze and object recognition tests [40]. The hAPP(751)_SL_ mice were purchased from, housed at, and treatment procedures were completed at JSW Life Sciences (Grambach, Austria).

For *in vitro* studies with primary microglial cultures, timed-pregnant Fisher 344 rats were purchased from Charles River Laboratories (Raleigh, NC). All animals were housed under a constant 12 hour light and dark cycle, and food and water were available *ad libitum*. All experiments were approved by the Virginia Commonwealth University Animal Care and Use Committee (AM10124) and conducted in strict accordance with guidelines set forth by the National Institutes of Health.

### Animal studies-treatment

The hAPP(751)SL mice were randomly assigned to one of 4 treatment groups: vehicle, dextromethorphan (DM) 15 mg/kg, DM 7.5 mg/kg, or apocynin 10 mg/kg. Starting at 4 months of age (±2 weeks), animals were either treated with saline (vehicle, n = 14), DM 15 mg/kg (n = 13), DM 7.5 mg/kg (n = 12), or apocynin 10 mg (n = 14) by oral gavage daily for 4 months.

### Morris Water Maze (MWM)

At the end of the 4 month treatment period, mice were trained in the MWM. The MWM tests took place in a black circular pool with a diameter of 100 cm filled with water and divided into four virtual quadrants. A transparent platform (diameter of 8 cm) was placed in the southwest quadrant of the pool. The walls surrounding the pool were marked with bold geometric symbols for spatial orientation. During behavioral testing, mice were placed in the pool and allowed to find the hidden platform. Ifthe mouse was unable to locate the platform, the investigatorguided the mouse. After each trial, mice were allowed to rest on the platform for 10–15 seconds and orient themselves. Mice performed three swimming trials per day for four consecutive days. During the trials, motion within the pool was detected with a computerized tracking system. These data were used to quantify swimming speed, escape latency (time, in seconds, for the mouse to find the hidden platform and escape the water), pathway (length traveled, in meters, before reaching the target), and abidance in the target quadrant (measured in percentage of the total trial time). Following the final trial on the fourth day, mice completed a ‘probe trial’ where the platform was removed and the number of crossings over the former platform position and abidance in the target quadrant were measured.

### Tissue collection

Following behavioral testing, animals were sacrificed and brain tissue was collected for further study. All mice were sedated using Isofluran inhalation before tissue collection. Mice were transcardially perfused with 0.9% saline and the brains were removed and divided into the right and left hemisphere. The left hemisphere was immediately processed for histology, while the right hemisphere was frozen on dry ice and stored at −80°C until use.

### TBARS assay

Lipid peroxidation in tissue samples was determined by the thiobarbituric acid reactive substances (TBARS) assay. Brain tissue was homogenized in 2.5% SDS with 5 mM butylated hydroxytoluene. 400 µL of this homogenate was mixed with 375 µL of 20% acetic acid, pH 3.5, and 225 µL of thiobarbituric acid (1.33%). The resulting mixture was incubated for 1 hour at 95°C. After incubation, 1 mL of 15∶1 butanol:pyridine was added and the mixture was centrifuged for 10 minutes at 4000 g. The amount of TBARS were determined by measuring the optical density of the organic layer at 535 nm and comparing the absorbance to a malondialdehyde (MDA) standard.

### Cellular fractionation: membrane preparation

Membrane fractions from both cell culture and tissue were isolated using differential centrifugation followed by lipid extraction [41]. Frozen brain tissue was suspended in fractionation buffer (FB; 20 mM HEPES, 250 mM sucrose, 1 mM EDTA, 10 ul/mL HALT protease inhibitor, 10 mM DTT, pH 7.5) and incubated for one minute at 37°C [42]. Brain samples were homogenized with a Teflon pestle 15 times and the resulting solution was centrifuged at 4°C, 600 g for 10 minutes. Supernatant was removed and an additional 2.5 mL of FB was added to the pellet. After resuspending the pellet, the samples were spun again at 4°C, 600 g for 10 minutes. The resulting supernatant was added to the first and centrifuged at 4°C, 15,000 g for 10 minutes. The supernatant from this spin was then centrifuged at 4°C, 100,000 g for 1 hour. The resulting pellet was solublized in 150 uL of 50 mM ammonium bicarbonate by vortexing. To this solution, 1 mL of 2∶1 TFE:Chloroform (freshly prepared) was added. Samples were placed on ice and vortexed for 1 minute every 10 minutes and then centrifuged at 4°C, 16,000 g for 10 minutes. The bottom chloroform layer (containing lipids) was removed, and the remaining top layer and insoluble phase were evaporated at 37°C in a SpeedVac. The resulting pellet was suspended in solublization buffer (SB; 8 M Urea, 25 mM TrisHCl, 2% SDS, 10 mM DTT, pH 7.5) and protein concentration was determined using the Coomassie Plus (Bradford) Protein Assay (ThermoScientific; Rockford, IL). The resulting samples were used to determine levels of NADPH oxidase activation by western blot, measuring the amount of p67 that had translocated to the membrane.

### Protein isolation: whole brain homogenate

From tissue samples, protein was isolated by suspending frozen tissue in 10 volumes of lysis buffer (Cytobuster Protein Extraction Reagent; EMD Chemicals; Darmstadt, Germany) with 10 µL/mL HALT protease inhibitor and 10 µL/mL EDTA. Samples were homogenized using a motorized pellet mixer and then centrifuged for 5 minutes at 5000 g. The protein concentration of the resulting supernatant was determined using a BCA protein assay (ThermoScientific; Rockford, IL).

### Immunoblotting

Protein samples were resolved by SDS-PAGE on 10% gels. Protein was then transferred to nitrocellulose membranes, blocked for 1 hour in 5% milk, and incubated overnight at 4°C in primary antibody (mouse anti-GAPDH, rabbit anti-p47-phox, or rabbit anti-p67-phox; Millipore; Temecula, CA). Blots were then probed with horseradish peroxidase-conjugated secondary antibodies and visualized using enhanced chemiluminescence (GE Healthcare; Piscataway, NJ).

### TNF α ELISA

The production and release of TNFα was measured using 100 µg/well of whole brain homogenate with a commercial enzyme-linked immunosorbent assay (ELISA) kit from R&D Systems (Minneapolis, MN), as described previously [43].

### Nitrotyrosine ELISA

The amount of nitrated proteins was measured using 100 µg/well of whole brain homogenate with a commercial enzyme-linked immunosorbent assay (ELISA) kit from Millipore (Temecula, CA), per the manufacturer protocol.

### Histology

#### Tissue fixation and sectioning

One hemisphere from each mouse brain was fixed by immersion in a solution of 4% paraformaldehyde in PBS (pH 7.4; freshly prepared) at 4°C for 24 hours. After fixation, brains were transferred to a 15% sucrose/PBS solution for 24 hours. Brains were then frozen in dry-ice cooled Isopentane and stored at −80°C until use. Frozen brains were sectioned into 15-10 µm thick sections per level (5 levels) starting at the level of the total appearance of the dentate gyrus and according to Paxinos and Franklin [44].

#### 6E10 and ThioflavinS double staining

The presence of amyloid depositions was visualized immunohistochemically using an anti-β-amyloid antibody directed against amino acids 1–17 of the human β-amyloid peptide (Signet Laboratories; Dedham, MA) with a Cy3 secondary antibody (Jackson Laboratories; Bar Harbor, ME). Additionally, tissue sections were stained with ThioflavinS to recognize beta-sheet structures. Briefly, sections were washed in H_2_O for 3 minutes and then placed in 1% ThioflavinS for 7 minutes. Sections were then washed in 80% ethanol and PBS before incubating in 1% hydrogen peroxide in methanol at room temperature for 15 minutes. Sections were then blocked using MOM-blocking reagent and MOM-diluent according to the manufacturer's protocol (MOM-Kit; Vector Labs; Burlingame, CA). After blocking, samples were incubated with 6E10 antibody (Signet Laboratories; Dedham, MA) for 30 minutes at room temperature, washed with PBS, and incubated in 10% non-immune goat normal serum for 60 minutes at room temperature. Sections were then washed and incubated with Cy3 goat anti-mouse antibody (Jackson Laboratories; Bar Harbor, ME) for 60 minutes in the dark at room temperature. Finally, the sections were washed in PBS and H_2_O before adding coverslips.

#### Measurement of amyloid deposition and plaque load

Measurement of 6E10 and ThioflavinS staining was done using Image-Pro Plus software (MediaCybernetics). Briefly, an area of interest (AOI) was measured encompassing both the hippocampus and cortex of each section. Within this AOI, stained objects were detected that were over a threshold level of intensity and a size of 8.75 µm^2^. A measurement of the area of each object, sum of stained area, and the number of objects was made in each AOI. Mean plaque size was calculated by dividing the sum area of plaques by the total number of plaques. The plaque area percentage was measured by dividing the sum area of plaques by the region area and multiplying the result by 100.

#### CD11b and synaptophysin immunohistochemistry

To determine microglial activation in brain slices, slices were stained with CD11b antibody. Synaptic density was visualized by staining with a synaptophysin antibody in separate brain slices. For both antibodies, frozen brain sections were washed for 10 minutes in PBS and then for 4 minutes in 1 mg/ml sodium-borohydrate in PBS. Sections were then washed and treated with 1% hydrogen peroxide in methanol at room temperature for 10 minutes. Non-specific binding was then blocked with 10% horse serum for 30 minutes and MOM-diluent (Mom-Kit; Vector Labs; Burlingame, CA) for 5 minutes. Sections were then incubated with anti-CD11b antibody (Serotec; Raleigh, NC) or anti-synaptophysin antibody (Thermo Fisher Scientific; Fremont, CA) for 1 hour at room temperature. Samples were incubated with blocking reagent (10% non-immuno goat-normal serum for CD11b and Vectastain Elite ABC Kit (Vector Labs; Burlingame, CA) for synaptophysin) for 20 minutes and room temperature and then washed with PBS. CD11b samples were then incubated with Cy 3 goat anti-rat antibody (Jackson Laboratories; Bar Harbor, ME), washed, and then stained with DAPI and methanol (Sigma Aldrich Chemical Co.; St. Louis, MO) for 15 minutes to stain cell nuclei. Sections were washed in 80% ethanol followed by H_2_O before adding coverslips. After primary antibody and blocking of synaptophysin-stained samples, samples were washed with PBS and incubated for 30 minutes with Vectastain ABC Reagent (Vector Labs; Burlingame, CA), washed, and developed for 18 minutes with HistoGreen (Linaris; Bettingen, Germany). Tissues were then washed in TBS and H_2_O and dehydrated with a graded alcohol series and xylol before adding coverslips.

### Measurement of microglia number *in vivo*

The number of microglia in each section was measured similarly to the protocol for 6E10 and ThioflavinS staining, except that the count only concentrated on CD11b staining that co-stained with the nucleus of the cell. Sections were co-stained with CD11b and DAPI, and cells were only counted in the AOI if their nucleus was within the name 10 µm thick section.

### Measurement of synaptic density *in vivo*

Synaptic density was also measured using Image-Pro Plus software (MediaCybernetics). Synapse number was counted at 1000-fold magnification from three images per region (CA1, CA3, and GDmb regions of the hippocampus). The total number of synapses was divided by the measured area (µm^2^) and averaged between the three images analyzed for each region.

### Cortical neuron-glia cultures

Rat cortical neuron-glia cultures were prepared using a previously described protocol [15]. Briefly, midbrain tissues were dissected from day 16/17 Fisher 344 rat embryos. Cells were dissociated via gentle mechanical trituration in minimum essential medium (MEM) and immediately seeded (5×10^5^/well) in poly D-lysine (20 µg/ml) pre-coated 24-well plates. Cells were seeded in maintenance media and exposed to the treatment media, as described previously [15]. Three days after seeding, the cells were replenished with 500 µL of fresh maintenance media. Cultures were treated 7 days after seeding.

### Microglia-enriched cultures

Primary enriched microglia cultures were prepared from the whole brains of day-old Fisher 344 rat pups, using the procedure described previously [45]. Briefly, after removing meninges and blood vessels, the brain tissue was gently triturated and seeded (5×10^7^) in 175 cm^3^ flasks. One week after seeding, the media was replaced. Two weeks after seeding, when the cells had reached a confluent monolayer of glial cells, microglia were shaken off and re-plated at 1×10^5^ in a 96-well plate. Cells were treated 24 hr after seeding the enriched microglia. Immunocytochemistry revealed less than 1% astrocyte or neuron contamination.

### Cell lines

The rat microglia HAPI cells were a generous gift from Dr James R. Connor [46] and were maintained at 37°C in DMEM supplemented with 10% FBS, 50 U/mL penicillin and 50 µg/mL streptomycin in a humidified incubator with 5% CO_2_/95% air.

### Beta-amyloid phagocytosis assay

The ability of cells to phagocytose β-amyloid peptide was measured using a protocol modified from Floden and Combs [47]. Fluorescently labeled β-amyloid 1–42 was prepared by adding 50 µL of 1% sterile ammonium hydroxide to lyophilized peptide, vortexing, adding 450 µL PBS, and incubating at 37°C for 1 week. Non-labeled β-amyloid 1–42 was suspended in PBS to give a 1 mM concentration, vortexed, and incubated at 37°C for one week. Aggregated β-amyloid (fluorescent or non-labeled) was aliquoted and stored at −20°C until use. To measure phagocytosis, primary microglia were seeded in a 96-well plate (0.5×10^5^ cells per well). 24 hours after seeding, cells were treated with 100 µM apocynin (solublized in DMSO) or control media for 30 minutes. Following pretreatment, cells were treated with control media, 100 µM apocynin, 2 µM non-labeled, aggregated β-amyloid 1–42, or apocynin and β-amyloid for 24 hours at 37°C. After 24 hours, media and treatments were removed, and cells were treated with control media, 100 µM apocynin, or cytochalasin D (control) for 30 minutes. To this treatment was then added 100 uL of 0.1 µM aggregated fluorescent β-amyloid. Plates were incubated for 6 hours to allow for phagocytosis of the fluorescent peptide, and then plates were read at 480 nm excitation, 520 nm emission.

### Superoxide Assay

Extracellular superoxide (O_2_^−^) production from microglia was determined as reported previously [48] by measuring the superoxide dismutase (SOD) inhibitable reduction of 2-(4-lodophenyl)-3-(4-nitrophenyl)-5-(2,4,-disulfophenyl)-2H-tetrazolium, monosodium salt (WST-1) [49], [50], [51]. The amount of SOD-inhibitable superoxide was calculated and expressed as percent of vehicle-treated control cultures.

### Hydrogen peroxide assay

Levels of hydrogen peroxide production in cell culture were determined as previously described, with slight modifications [52]. Briefly, cells were seeded in a 96-well plate (0.75×10^5^ cells per well) and incubated for 24 hours at 37°C. Cells were then washed once with warm HBSS, and then 50 µL of HBSS was added to each well, followed by 50 µL of control (HBSS) or treatment. To each well, 100 µL of assay mix (200 µM homovanillic acid, 10 U/mL horseradish peroxidase, 2 mM HEPES, pH 7.5) with or without catalase (10,000 U/mL), was added. Cells were incubated for 3 hours at 37°C. Following incubation, 16 µL of stop solution (0.1 M glycine, pH 10) was added to each well, and the plates were read at 321 nm excitation, 421 nm emission. Results are calculated as catalase-inhibitable florescence and reported as a percent of control values.

### Cell survival (MTT) assay

Cell survival was measured using thiazole blue (MTT) to evaluate metabolic viability of cells [53]. Microglia enriched cultures (1×10^5^ cells per well in a 96-well plate) were pretreated with 100 µM apocynin or control media for 30 minutes, and then treated with 100 ng/mL LPS, 2 µM staurosporine, 2 µM Aβ, DMSO, or control media for 24 hours. After the 24 hour incubation, 5 mg/mL MTT was added to cells in a 96-well plate for a final concentration of 0.1 mg/mL. Cells were then incubated for 90 minutes at 37°C. MTT and culture media were removed from the wells and 100 µL of DMSO was added to each well. The plate was then placed on an orbital shaker for 30 minutes and the absorbance was read at 550 nm.

### Microglia Cell Number *in vitro*

Microglial cell number was measured by taking microglia cell counts from mixed neuron-glia cultures treated for 24 hours with 10 ng/mL LPS or 2 µM Aβ with or without 100 µM apocynin. After treatment, cells were fixed in 3.7% formaldehyde, washed once with PBS, and treated with 1% hydrogen peroxide. Cells were then washed three times with PBS and blocked for one hour in PBS with 1% bovine serum albumin, 0.4% Triton X-100 and 4% goat serum. Plates were then incubated overnight at 4°C in a 1∶1000 dilution of anti-IBA-1 antibody (Wako Pure Chemical Industries, Ltd., Richmond, VA) in Dako antibody diluent (DAKO, Capinteria, CA). After incubation with primary antibody, cells were washed three times and incubated with Vectastain ABC Kit reagents according to the manufacturer's instructions (Vector Laboratories, Burlingame, CA). Images were taken on an AxioCam MRc5 imaging system (Carl Zeiss MicroImaging, Thornwood, NY). Cell numbers were quantified by counting nine representative areas per well in a 24 well plate at 100X magnification (an average of numbers counted by at least 2 individuals is reported).

### Statistical Analysis

Group differences in the behavioral tests were calculated using a parametric ANOVA with a Bonferroni's multiple comparison post-hoc test or a non-parametric Kruskal Wallis ANOVA with a Dunn's multiple comparison test if Gaussian distribution was missing. For assessments of learning deficits, a two-way ANOVA was used followed by Bonferroni's multiple comparison test. For *in vitro* studies, significance was calculated using a one-way ANOVA followed by a Bonferroni post-hoc test. The treatment groups are expressed as the mean ± SEM. A value of p<0.05 was considered statistically significant.

## Results

### Apocynin reduces plaque size in the cortex and hippocampus of hAPP(751)_SL_ mice

**Figure 1:**
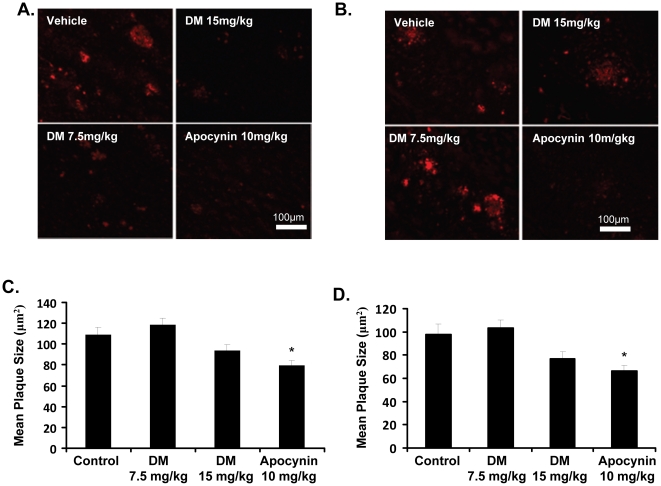
Apocynin reduces plaque size in the cortex and hippocampus of hAPP(751)_SL_ mice. Mice were treated daily with 15 mg/kg dextromethorphan (DM), 7.5 mg/kg DM, or 10 mg/kg apocynin for four months. The size of β-amyloid plaques was measured for each group and compared to control, vehicle-treated animals. Representative images show 6E10 staining of β-amyloid protein for each group in the cortex (A) and hippocampus (B), respectively. Quantification of plaque size the cortex (C) and hippocampus (D) revealed that only apocynin significantly decreased the size of plaques, compared to vehicle. DM, at either dose, did not alter plaque size in the cortex or the hippocampus. Plaque size was determined as the absolute plaque area divided by the absolute plaque number. *p<0.05 vs. vehicle, 1-way ANOVA with Bonferroni post-hoc test.

Brain slices from each group (vehicle, 15 mg/kg DM, 7.5 mg/kg DM, 10 mg/kg apocynin) were stained for two markers of Aβ deposition: 6E10 (measuring all Aβ peptide) and thioflavin S (measuring β-sheets of Aβ). This allowed for the measurement of plaque number, mean plaque size, and the percentage of area occupied by plaques in both the cortex and hippocampus. Using 6E10 staining, both the cortex and hippocampus display reduced plaque size in apocynin treated animals, compared to vehicle-treated controls (p<0.05; Figure 1). DM, at either dose, did not produce any significant reduction in plaque size. No significant differences were observed in plaque number or percentage area in the hippocampus or cortex with any of the treatments (data not shown). Additionally, ThioflavinS staining of β-sheets revealed no differences between vehicle, DM, and apocynin-treated hAPP(751)_SL_ mice (data not shown).

### Apocynin reduces the number of microglia in the cortex of hAPP(751)_SL_ mice

**Figure 2:**
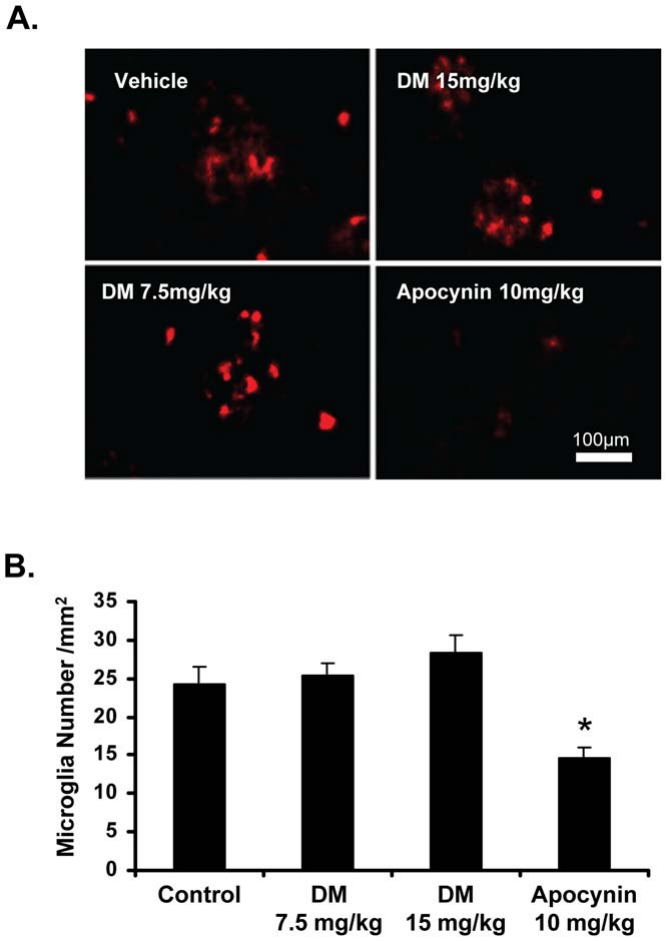
Apocynin reduces the number of microglia in the cortex of hAPP(751)_SL_ mice. Mice were treated daily with 15 mg/kg dextromethorphan (DM), 7.5 mg/kg DM, or 10 mg/kg apocynin for four months. The number of microglia was then counted for each group by staining with anti-CD11b antibody and each treatment group was compared to control. CD11b-stained microglia were only counted if they corresponded to a DAPI stained nuclei (data not shown). Representative images from each group of the stained microglia are shown in panel (A). Apocynin reduced the number of microglia in the cortex of hAPP(751)_SL_ mice, whereas neither dose of DM reduced microglia number (B). *p<0.05 vs vehicle, 1-way ANOVA with Bonferroni post-hoc test.

The number of microglia in both the cortex and hippocampus of hAPP(751)_SL_ mice with DM or apocynin treatment was counted using CD11b immunoreactivity. Decreases in the number of microglia in the cortex was observed in mice treated with 10 mg/kg apocynin (p<0.05; Figure 2). No changes were seen in the hippocampus (Table S1).

### Neither dextromethorphan nor apocynin improve behavioral deficits in hAPP(751)_SL_ mice

Behavioral deficits were measured in hAPP(751)_SL_ mice by performance in the Morris Water Maze (MWM) through 3 daily trials over 4 consecutive days after 4 months of treatment with vehicle (0.9% saline), 15 mg/kg DM, 7.5 mg/kg DM, or 10 mg/kg apocynin. Overall performance in the MWM was determined by escape latency (seconds) and swimming path (meters). A downward trend was observed within groups on subsequent days (data not shown), indicating that each treatment group was able to learn and improve overall performance. No significant changes were seen in escape latency or swimming path between groups on any of the days (Table S2). A trend in improvement in the swimming path was observed between vehicle and apocynin treated mice on day 2 of the 4 day test (ANOVA p = 0.084; t-test p = 0.024), although this was not observed on any of the other days or other MWM tests. At the end of the testing period (day 4), the hidden platform was removed from the pool and measures of abidance in the target quadrant and the number of target crossing were taken. No significant changes were observed between groups for either measurement (Table S2).

### Neither dextromethorphan nor apocynin alter synapse density in the hippocampus of hAPP(751)_SL_ mice

Synapse density was measured in the CA1, CA3, and GDmb regions of the hippocampus with synaptophysin immunoreactivity. Neither DM nor apocynin altered synapse density of any of the regions examined (data not shown). This is consistent with the lack of behavioral changes seen in hAPP(751)_SL_ mice treated with apocynin and DM.

### NADPH oxidase activation, TNFα, lipid peroxidation, and nitrotyrosine levels in hAPP(751)_SL_ mouse brains are low, and unaffected by treatment with dextromethorphan or apocynin

The ability of DM and apocynin to reduce NADPH oxidase activation in hAPP(751)_SL_ *mice* was measured by western blot analysis of translocation of the p67^PHOX^ cytosolic protein to the membrane, as previously reported [54]. Notably, there were low levels of NADPH oxidase activation which was not modified by either apocynin or DM (data not shown). Similarly the levels of TNFα (as measured by ELISA) were not altered with DM or apocynin treatment (data not shown), where levels of TNFα in vehicle-treated controls were negligible (∼100 pg/mg of total protein), indicating low basal levels of TNFα in hAPP(751)_SL_ mice at 8 months of age. Levels of nitro-tyrosine were also measured via ELISA, where again levels of nitrotyrosine in vehicle treated controls (basal levels) were very low (1.32 µg/mL; Table S3), further supporting an absence of oxidative stress.

Brain homogenates from each group (vehicle, 15 mg/kg DM, 7.5 mg/kg DM, 10 mg/kg apocynin) were used to measure the levels of lipid peroxidation using a TBARS assay. Levels of malondialdehyde (MDA) from each group were approximately 2.5 µM, suggesting low levels of oxidative stress. This is particularly interesting, as lipid peroxidation has previously been reported to increase significantly in post-mortem analysis of preclinical [55] and diagnosed AD [56], [57] brain. No statistically significant changes were observed in the levels between groups (data not shown), indicating that the treatments (DM or apocynin) to not alter levels of oxidative stress in hAPP(751)_SL_ mice.

Thus, neuroinflammation and oxidative stress were not readily apparent at 8 months of age in the hAPP(751)SL mice tested, which may explain why the known NADPH oxidase inhibitors failed to reduce these parameters. Together, these findings also indicate that although apocynin inhibited microglial number and plaque formation, it is very likely that it did so through mechanisms that are independent of anti-inflammatory and antioxidant properties. In addition, these findings also indicate that Aβ plaque load, microglia number, and learning deficits may occur independently of neuroinflammation and oxidative stress.

### Apocynin & dextromethorphan reduce Aβ-induced superoxide and are neuroprotective *in vitro*

**Figure 3:**
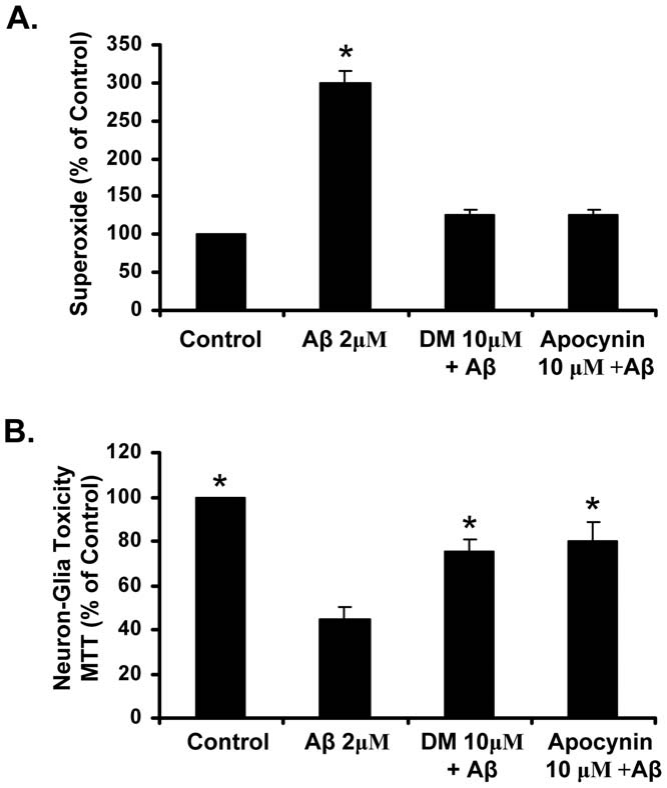
Apocynin reduces NADPH oxidase activation and is neuroprotective *in vitro*. (A) Enriched microglia cultures were treated with media alone (Control), apocynin (10 µM), Dextromethorphan (DM, 10 µM), Aβ (2 µM), Apocynin + Aβ, and DM + Aβ. The production of extracellular superoxide was measured by the superoxide dismutase (SOD)-inhibitable reduction of tetrazolium salt, WST-1 at 30 minutes post-treatment. Results are mean ± SEM. Data are from four separate experiments. *p<0.05, compared with control cultures. (B) Apocynin and DM protect against Aβ-induced toxicity in cortical neuron-glia cultures.) Cortical neuron-glia cultures were treated with media alone (Control), Apocynin (10 µM), Dextromethorphan (DM, 10 µM), Aβ (2 µM), Apocynin + Aβ, and DM + Aβ. Toxicity was assessed by MTT 7 days later. Graphs show the results expressed as percentage of the control cultures and are the mean ± SEM from three independent experiments in triplicate. * p<0.05, control compared to treatment.

To confirm that both apocynin and DM were capable of inhibiting NADPH oxidase at all, we next tested their ability to reduce the production of extracellular ROS and neurotoxicity in response to Aβ. Both apocynin and DM were able to reduce the production of Aβ-induced extracellular superoxide to nearly control levels in primary microglia cultures (Figure 3) and ameliorate Aβ-induced neurotoxicity in cortical mixed-neuron-glia cultures (Figure 3). Thus, in the presence of microglial NADPH oxidase activation *in vitro*, both compounds are able to reduce extracellular ROS and cellular damage. These findings further support that the inability of either DM or apocynin to reduce measures of oxidative stress and synaptic density may have been due to a lack of activation of NADPH oxidase and neuroinflammation in hAPP(751)_SL_ mice at 8 months.

### Apocynin reduces H_2_O_2_, but does not reverse Aβ-induced decreases in phagocytosis *in vitro*

**Figure 4:**
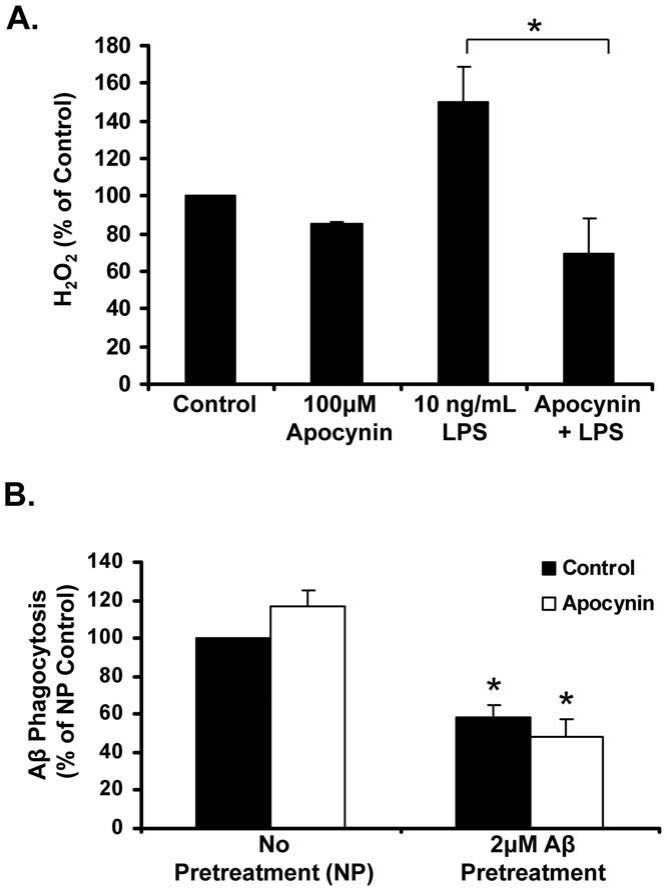
Apocynin regulates microglial H_2_O_2_ production, but not Aβ phagocytosis. (A) Apocynin attenuates LPS-induced hydrogen peroxide (H_2_O_2_), as predicted. Microglia-enriched cultures were treated with Hank's balanced salt solution (HBSS), or HBSS with LPS (10 ng/mL), apocynin (100 µM), or the combination of apocynin (100 µM) and LPS for 3 hours. The level of H_2_O_2_ was then measured in each group and compared to control levels. Apocynin does significantly reduce LPS-induced increases in H_2_O_2,_ returning levels to control values. *p<0.05 vs. control; #p<0.05 vs. LPS, 1-way ANOVA with Bonferroni post-hoc test. (B) Pre-treatment with 2 µM Aβ significantly reduces phagocytosis of fluorescent Aβ, and apocynin does not act to reverse this decrease. Microglia-enriched cultures were treated with control media, or media with β-amyloid (Aβ; 2 µM), apocynin (100 µM), or the combination of apocynin (100 µM) and Aβ (2 µM) for 24 hours. Fluorescently labeled Aβ (final concentration 0.1 µM) was then added to each well, and incubated with the cells for 6 hours to allow for phagocytosis of the fluorescent protein. The amount of phagocytosis of fluorescent Aβ was measured for each group and compared to control levels. *p<0.05 vs control, 1-way ANOVA with Bonferroni post-hoc test.

We confirmed that apocynin was working as predicted by demonstrating that 30 minute pretreatment with apocynin will attenuate LPS-induced H_2_O_2_ production (Figure 4). To test a possible mechanism for the *in vivo* observation of decreased plaque size with apocynin treatment, the capability of microglial to phagocytize fluorescently labeled Aβ was tested in microglial-enriched primary cell cultures. Pre-treatment of cells with 2 µM Aβ for 24 hours prior to the addition of the fluorescent Aβ significantly reduces the phagocytosis capacity of microglia by 41% (p<0.05) (Figure 4). Co-treatment with 100 µM apocynin did not reverse the Aβ-induced decreases in fluorescent Aβ phagocytosis (Figure 4), supporting that superoxide and reactive oxygen species (e.g. ROS) do not mediate the Aβ-induced loss of phagocytic function. Thus, while loss of microglial phagocytic function has been implicated as a key component to the development of plaques and AD progression [58], [59], apocynin failed to ameliorate this response in vitro, indicating it is an unlikely mechanism in the *in vivo* effects on plaque size.

### Apocynin attenuates LPS-induced increases in cytokine production *in vitro*

**Figure 5:**
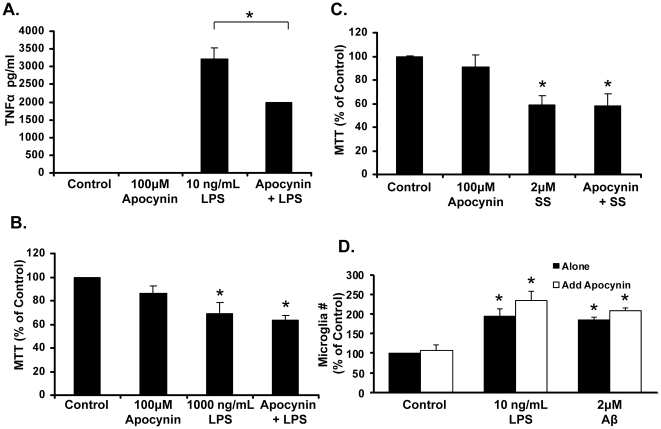
Apocynin ameliorates LPS-induced TNFα production, but has no effect on microglial cell death or cell number *in vitro*. (A) Microglia-enriched cultures were treated with control media, lip polysaccharide (LPS; 10 ng/mL), and/or Apocynin (100 µM) for 24 hours. Tumor necrosis factor alpha (TNFα) levels in the supernatant were measured via ELISA. LPS (10 ng/mL) significantly increased levels of TNFα and pre-treatment with 100 µM Apocynin significantly reduced the amount of TNFα released by microglia. *p<0.05 vs. control; #p<0.05 vs. LPS, 1-way ANOVA with Bonferroni post-hoc test. (B) Apocynin does not protect against inflammation-induced cell death. Microglia-enriched cultures were treated with control media, 1000 ng/mL LPS, 100 µM Apocynin, or LPS and apocynin for 24 hours. After incubation, cell survival was measured with the MTT assay. 100 ng/mL LPS significantly reduced microglial cell survival (through inflammation-induced cell death), which is not rescued by apocynin). (C) Microglia-enriched cultures were treated with control media, 2 µM staurosporine (SS), 100 µM Apocynin, or SS and apocynin for 24 hours. After incubation, cell survival was measured with the MTT assay. Data show that 2 µM SS significantly reduces microglial cell survival (through apoptosis), and is not reversed by the addition of apocynin. *p<0.05 vs control,1-way ANOVA with Bonferroni post-hoc test. (D) Apocynin does not alter Aβ or LPS-induced increases in microglia number *in vitro*. Mixed neuron-glia cultures were treated with 2 µM Aβ, 10 ng/mL LPS and/or 100 µM apocynin for 24 hours. Cultures were then fixed and stained with IBA-1 antibody for microglia. The number of microglia was then counted in 9 representative areas per well. The number of microglia was significantly increased in cultures treated with LPS (196% of control) and Aβ (186% of control). Apocynin treatment did not prevent the increase in cell count caused by LPS or Aβ. Apocynin alone caused no significant change in cell count. *p<0.05 vs control, 1-way ANOVA with Bonferroni post-hoc test.

We then focused *in vitro* analyses specifically on apocynin, which reduced both plaque size and microglial number *in vivo*. Specifically, we next addressed whether apocynin was able to ameliorate a generalized pro-inflammatory response from microglia. The pro-inflammatory cytokine response of primary microglia-enriched cultures was tested by measuring levels of TNFα following treatment with 10 ng/mL LPS and/or 100 µM apocynin. LPS significantly increased levels of TNFα at 24 hours after treatment (to 2907 pg/mL), and apocynin was able to significantly reduce this response (reduced to 1952 pg/mL), although levels did not return to that of control (4 pg/mL) (Figure 5) (p<0.05). These data confirm that if TNFα levels are elevated, in the very least, apocynin is able to reduce them *in vitro*.

### Apocynin does not inhibit apoptotic or inflammation-induced cell death in microglia *in vitro*

We also considered that the reduction of microglial cell number in the brains of hAPP(751)_SL_ mice could be a result of increases microglial cell death or a reduction in proliferation. To test microglial cell survival in response to a number of toxic stimuli, and the effect that apocynin has on this response, primary microglia-enriched cultures were treated with 2 µM Aβ, 1000 ng/mL LPS (to cause inflammation-induced cell death), or 2 µM staurosporine (to induce apoptotic cell death) in the presence and absence of 100 µM apocynin. Neither Aβ, apocynin, nor the combination reduced cell survival *in vitro* (data not shown). Both LPS (Figure 5) and staurosporine (Figure 5) significantly reduced cell survival with 24 hours of treatment, but apocynin is not capable of preventing either inflammation-induced or apoptotic cell death (p<0.05) (Figure 5).

### Apocynin does not alter Aβ or LPS-induced increases in microglia number *in vitro*

To look at the effect of Aβ and/or apocynin on microglial cell proliferation (to possibly account for the reduction in microglial number observed *in vivo*), mixed neuron-glia cultures were treated for 24 hours with 2 uM Aβ, 10 ng/mL LPS and/or 100 µM apocynin and microglia were stained with IBA-1 antibody, and counted. Both LPS and Aβ significantly increased the number of microglia in the mixed neuron-glia culture (196% and 186% of control, respectively; p<0.05) (Figure 5). Apocynin, however, had no significant effect on the number of IBA-1 stained microglia in LPS or Aβ-treated mixed neuron-glia cultures.

## Discussion

Accumulating evidence indicates that the ideal therapeutic window for anti-inflammatory treatment targeting neurotoxic microglial activation may be early in the neurodegenerative process [26], highlighting a role for prevention. Microglial NADPH oxidase has been implicated in the progressive nature of AD through the chronic production of ROS in response to Aβ and/or neuron damage and the amplification of pro-inflammatory factors, such as TNFα. Here, we used an *in vivo/in vitro* approach to test the hypothesis that inhibition of NADPH oxidase reduces microglia-mediated neuropathology (neuroinflammation, oxidative stress, and neuron damage) and behavioral symptoms (learning and memory deficits) associated with AD. Specifically, using the hAPP(751)_SL_ transgenic mouse model of AD, the ability of chronic administration (4 month) of two NADPH oxidase inhibitors (apocynin and DM) to prevent toxic microglial activation, reduce plaque size, preserve neuron function, and attenuate cumulative learning and memory deficits was tested.

Importantly, this study also addressed the utility of the hAPP(751)_SL_ transgenic mouse model for testing anti-inflammatory compounds. The hAPP(751)_SL_ mice over-express human APP Swiss and London mutations, with elevated expression in neurons throughout the brain, pronounced expression in the hippocampus, and little expression the periphery [60], [61], [62]. Amyloid depositions occur as plaques and begin at 3 to 4 months in hAPP(751)_SL_ mice, with accumulation in the hippocampus commencing at 7 months [60], [61], [62]. Using this defined window of plaque deposition, we sought to prevent neuropathology and behavioral deficits by administering the drugs early, from 4–8 months of age, before significant damage had occurred. Despite the high level of Aβ, accumulation of Aβ protein deposits, and behavioral deficits associated with this model [60], [61], [62], we found that at 8 months hAPP(751)_SL_ mice showed little evidence of neuroinflammation and oxidative stress in saline control animals, as TNFα protein, lipid peroxidation, protein nitration, and NADPH oxidase activation were low, making reduction by any inhibitors improbable. We were intrigued by these findings, as there is a well established link between Aβ and neuroinflammation/oxidative stress [19]. Further, post-mortem analysis of AD brains reveal microglia clustered around plaques combined with high levels of oxidative stress and neuroinflammation [63], [64], including activation of NADPH oxidase [1]. However, recent reports reveal distinct differences in murine AD models when compared to the human disease that are consistent with our findings. For example, the activation of complement, which is absent in mouse models and present in human disease, has been strongly implicated in the cross-species difference in neuroinflammation [65]. Yet, recent reports employing a slightly different murine model, aged (14 month) R1.40 mice, show that NADPH oxidase is activated in these aged mice and that this response can be modified by ibuprophin [5]. While this study reported significant effects on plaque load, microglial activation, and indicators of oxidative stress that were modified by ibuprophin [5], the effect on cytokines, neuron damage, and behavioral deficits were not discussed. Thus, it remains possible that with significant aging (perhaps at 14 months) there would be more pronounced evidence of NADPH oxidase activation and neuroinflammation in the hAPP(751)_SL_ model also.

However, despite the lack of evidence for NADPH oxidase-induced pathology *in vivo* and the consequent inability of either compound tested to regulate the enzyme complex's low function in hAPP(751)_SL_ mice, apocynin (and not DM) treatment reduced microglial number (Figure 2) and Aβ plaque size (Figure 1) *in vivo*. While *in vitro* analysis employed the use of immature cells and cell lines, the data revealed that apocynin had no effect on microglia cell death (Figure 5), nor microglial increases in neuron-glial cultures treated with LPS or Aβ (Figure 5). Together, these results suggest that the reduction of microglia number in vivo may not be due to direct effects of apocynin on microglial number, but may instead occur through effects on the deposition, such as APP processing/amyloidgenesis, Aβ aggregation, Aβ transport, or degradation of Aβ. Rather, we speculate that perhaps the reduction in microglial number by apocynin may be driven by the reduced plaque size.

The reduction in cortex and hippocampus plaque size conferred by apocynin could be the consequence of a number of processes, including plaque phagocytosis, deposition, degradation, or APP processing and transport. As loss of microglial phagocytic function has been implicated as a key component to the development of plaques and AD progression [58], [59], we next tested the ability of apocynin to regulate Aβ fibril phagocytosis. Our results indicate that treatment of primary microglia cultures with high doses (2 µM) of unlabeled, fibrilized Aβ for 24 hours reduced the ability of microglia to phagocytize fluorescent Aβ after the unlabeled ligand was washed away, supporting that high levels of Aβ may reduce microglial phagocytosis. However, our data also indicate that apocynin does not modify Aβ phagocytosis in any of the conditions tested. Therefore, the reduction in plaque size observed in hAPP(751)SL mice with apocynin treatment are independent the microglial functions tested here.

Another interesting finding emphasized by this work is the disconnect between plaque size and memory deficits in the hAPP(751)SL mice. While apocynin was able to reduce plaque size (Figure 1) and microglial number (Figure 2) *in vivo*, there were no significant effects on behavior or synaptic density (synaptophysin staining). This was unexpected, as apocynin has been shown to protect against behavioral deficits linked to chronic brain hypoxia [66] and presumably the behavior loss in hAPP(751)SL mice is due to Aβ deposition. As neuronal damage and behavioral deficits in the hAPP(751)SL model peak around 14 months of age, aging may again be necessary to acquire more AD-relevant pathology for this model. Alternatively, it is also possible that a reduction of greater than 50% of the plaque size is necessary to impact synaptic plasticity and behavior.

The *in vitro* component of this study demonstrated that both DM and apocynin attenuate Aβ-induced extracellular superoxide (O_2_^•-^) production in primary microglia cultures (Figure 3) and protect against Aβ-induced toxicity in cortical mixed neuron-glia cultures (Figure 3), as expected. In addition, further *in vitro* analysis with apocynin including several functional positive controls revealed that apocynin reduced H_2_O_2_ production (Figure 4) and LPS-induced cytokine production (Figure 5), demonstrating its established anti-inflammatory properties as expected. Furthermore, apocynin and associated metabolites readily reach the brain, where they have demonstrated properties such as NADPH oxidase inhibition, neuroprotection, and anti-inflammatory properties in other CNS disease models, such as hypoxia [66]. This further supports the premise that NADPH oxidase activation was not present in hAPP(751)SL mice at this time.

In summary, apocynin treatment for 4 months in hAPP(751)SL mice reduced plaque size and microglial number, resulting in brains that resembled younger mice. *In vitro* analysis confirmed that apocynin reduced Aβ toxicity in mixed cortical neuron-glia cultures, and H_2_O_2_, O_2_^•-^, and TNFα production in primary microglia cultures. However, *in vivo* analysis revealed no effects for apocynin on synaptophysin (indicative of subleathal neuronal damage) or behavioral measures of learning and memory. In fact, upon further analysis, it was apparent that 8 month old hAPP(751)SL mice presented low levels of neuroinflammation and oxidative stress, which not surprisingly, was unaffected by apocynin. Additional *in vitro* study indicated that apocynin failed to affect microglial death, proliferation, and phagocytosis, indicating that the microglia number and plaque size reduction *in vivo* likely occur through unknown mechanisms that are independent of apocynin's anti-inflammatory characteristics. Together, these findings suggest that apocynin is a unique NADPH oxidase inhibitor with anti-β amyloid traits, supporting its possible use as a novel and preventative therapeutic compound for early AD.

## Supporting Information

Table S1 **Microglial number in the hippocampus of hAPP(751)_SL_ mice.** The number of microglia was measured in hAPP(751)SL mice (Vehicle, DM 15 mg/kg, DM 7.5 mg/kg, and Apocynin 10 mg/kg) following 4 months of treatment. While the total number of microglia decreased significantly in the cortex (Figure 2), the number in the hippocampus showed only a trend toward a decrease in the numbers, as seen by staining with anti-CD11b antibody (statistical significance was tested with 1-way ANOVA with Bonferroni post-hoc test).
(DOC)

Table S2 **Behavioral measures in hAPP(751)_SL_ mice.** hAPP(751)SL mice (Vehicle, DM 15 mg/kg, DM 7.5 mg/kg, and Apocynin 10 mg/kg) were behaviorally tested using the Morris Water Maze. Measures of time learning (escape latency), length learning (length of swimming path), abidance in the target quadrant (% of total time), and number of target crossings were made for each animal, and values were compared to vehicle treated animals and tested for statistical significance (1-way ANOVA with Bonferroni post-hoc test, where applicable). No statistically significant changes were observed for any of the measurements, although trends toward improvement were seen in some tests. * p-value vs. vehicle is displayed when there was a trend (p<0.1) in ANOVA data.” Where trends were seen (ANOVA p<0.1) the p-value versus control is shown.
(DOC)

Table S3 **The effect of apocynin on levels of nitro-tyrosine in hAPP(751)SL mice.** Following 4 months of treatment with Vehicle, DM 15 mg/kg, DM 7.5 mg/kg, or Apocynin 10 mg/kg, total protein was isolated from the brains of hAPP mice and the levels of nitrotyrosine were determined by ELISA (Millipore). No significant changes were observed in the levels of nitro-tyrosine between vehicle and treated mice. However, it is of note that the levels are very low, even in the vehicle treated hAPP transgenic mice (statistical significance was tested with 1-way ANOVA).
(DOC)

## References

[1] Block ML (2008). NADPH oxidase as a therapeutic target in Alzheimer's disease.. BMC Neurosci.

[2] Imbimbo BP, Solfrizzi V, Panza F (2010). Are NSAIDs useful to treat Alzheimer's disease or mild cognitive impairment?. Front Aging Neurosci.

[3] Babior BM (2000). Phagocytes and oxidative stress.. Am J Med.

[4] Qin L, Liu Y, Wang T, Wei SJ, Block ML (2004). NADPH oxidase mediates lipopolysaccharide-induced neurotoxicity and proinflammatory gene expression in activated microglia.. J Biol Chem.

[5] Wilkinson BL, Cramer PE, Varvel NH, Reed-Geaghan E, Jiang Q (2010). Ibuprofen attenuates oxidative damage through NOX2 inhibition in Alzheimer's disease.. Neurobiol Aging.

[6] Block ML, Hong JS (2005). Microglia and inflammation-mediated neurodegeneration: multiple triggers with a common mechanism.. Prog Neurobiol.

[7] Shimohama S, Tanino H, Kawakami N, Okamura N, Kodama H (2000). Activation of NADPH oxidase in Alzheimer's disease brains.. Biochem Biophys Res Commun.

[8] Wu DC, Teismann P, Tieu K, Vila M, Jackson-Lewis V (2003). NADPH oxidase mediates oxidative stress in the 1-methyl-4-phenyl-1,2,3,6-tetrahydropyridine model of Parkinson's disease.. Proc Natl Acad Sci U S A.

[9] Rogers J, Luber-Narod J, Styren SD, Civin WH (1988). Expression of immune system-associated antigens by cells of the human central nervous system: relationship to the pathology of Alzheimer's disease.. Neurobiol Aging.

[10] McGeer PL, Itagaki S, Tago H, McGeer EG (1987). Reactive microglia in patients with senile dementia of the Alzheimer type are positive for the histocompatibility glycoprotein HLA-DR.. Neurosci Lett.

[11] Cagnin A, Brooks DJ, Kennedy AM, Gunn RN, Myers R (2001). In-vivo measurement of activated microglia in dementia.. Lancet.

[12] Yankner BA (1989). Amyloid and Alzheimer's disease–cause or effect?. Neurobiol Aging 10: 470-471; discussion.

[13] Yankner BA, Duffy LK, Kirschner DA (1990). Neurotrophic and neurotoxic effects of amyloid beta protein: reversal by tachykinin neuropeptides.. Science.

[14] Combs CK, Johnson DE, Karlo JC, Cannady SB, Landreth GE (2000). Inflammatory mechanisms in Alzheimer's disease: inhibition of beta-amyloid-stimulated proinflammatory responses and neurotoxicity by PPARgamma agonists.. J Neurosci.

[15] Qin L, Liu Y, Cooper C, Liu B, Wilson B (2002). Microglia enhance beta-amyloid peptide-induced toxicity in cortical and mesencephalic neurons by producing reactive oxygen species.. J Neurochem.

[16] Sasaki A, Yamaguchi H, Ogawa A, Sugihara S, Nakazato Y (1997). Microglial activation in early stages of amyloid beta protein deposition.. Acta Neuropathol (Berl).

[17] Meda L, Cassatella MA, Szendrei GI, Otvos L, Baron P (1995). Activation of microglial cells by beta-amyloid protein and interferon-gamma.. Nature.

[18] Li MSM, Ohnishi K, Ichimori Y (1996). beta-Amyloid protein-dependent nitric oxide production from microglial cells and neurotoxicity Brain Res.

[19] Wilkinson BL, Landreth GE (2006). The microglial NADPH oxidase complex as a source of oxidative stress in Alzheimer's disease.. J Neuroinflammation.

[20] Dheen ST, Jun Y, Yan Z, Tay SS, Ang Ling E (2004). Retinoic acid inhibits expression of TNF-alpha and iNOS in activated rat microglia.. Glia.

[21] Griffin WS, Sheng JG, Royston MC, Gentleman SM, McKenzie JE (1998). Glial-neuronal interactions in Alzheimer's disease: the potential role of a 'cytokine cycle' in disease progression.. Brain Pathol.

[22] Reed-Geaghan EG, Savage JC, Hise AG, Landreth GE (2009). CD14 and toll-like receptors 2 and 4 are required for fibrillar A{beta}-stimulated microglial activation.. J Neurosci.

[23] Wilkinson BL, Landreth GE (2006). The microglial NADPH oxidase complex as a source of oxidative stress in Alzheimer's disease.. J Neuroinflammation.

[24] Levesque S, Wilson B, Gregoria V, Thorpe LB, Dallas S (2010). Reactive microgliosis: extracellular micro-calpain and microglia-mediated dopaminergic neurotoxicity.. Brain.

[25] Gao HM, Liu B, Zhang W, Hong JS (2003). Critical role of microglial NADPH oxidase-derived free radicals in the in vitro MPTP model of Parkinson's disease.. Faseb J.

[26] Block ML, Zecca L, Hong JS (2007). Microglia-mediated neurotoxicity: uncovering the molecular mechanisms.. Nat Rev Neurosci.

[27] Block ML, Hong JS (2007). Chronic microglial activation and progressive dopaminergic neurotoxicity.. Biochem Soc Trans.

[28] Albrecht-Goepfert E, Schempp H, Elstner EF (1998). Modulation of the production of reactive oxygen species by pre-activated neutrophils by aminoadamantane derivatives.. Biochem Pharmacol.

[29] Wu HM, Tzeng NS, Qian L, Wei SJ, Hu X (2009). Novel neuroprotective mechanisms of memantine: increase in neurotrophic factor release from astroglia and anti-inflammation by preventing microglial activation.. Neuropsychopharmacology.

[30] Cordle A, Landreth G (2005). 3-Hydroxy-3-methylglutaryl-coenzyme A reductase inhibitors attenuate beta-amyloid-induced microglial inflammatory responses.. J Neurosci.

[31] Li G, Cui G, Tzeng NS, Wei SJ, Wang T (2005). Femtomolar concentrations of dextromethorphan protect mesencephalic dopaminergic neurons from inflammatory damage.. FASEB J.

[32] Liu Y, Qin L, Li G, Zhang W, An L (2003). Dextromethorphan protects dopaminergic neurons against inflammation-mediated degeneration through inhibition of microglial activation.. J Pharmacol Exp Ther.

[33] Zhang W, Wang T, Qin L, Gao HM, Wilson B (2004). Neuroprotective effect of dextromethorphan in the MPTP Parkinson's disease model: role of NADPH oxidase.. Faseb J.

[34] Gao HM, Liu B, Zhang W, Hong JS (2003). Novel anti-inflammatory therapy for Parkinson's disease.. Trends Pharmacol Sci.

[35] Rosi S, Vazdarjanova A, Ramirez-Amaya V, Worley PF, Barnes CA (2006). Memantine protects against LPS-induced neuroinflammation, restores behaviorally-induced gene expression and spatial learning in the rat.. Neuroscience.

[36] Liu SL, Li YH, Shi GY, Tang SH, Jiang SJ (2009). Dextromethorphan reduces oxidative stress and inhibits atherosclerosis and neointima formation in mice.. Cardiovasc Res.

[37] Van den Worm E, Beukelman CJ, Van den Berg AJ, Kroes BH, Labadie RP (2001). Effects of methoxylation of apocynin and analogs on the inhibition of reactive oxygen species production by stimulated human neutrophils.. Eur J Pharmacol.

[38] Gao HM, Liu B, Zhang W, Hong JS (2003). Synergistic dopaminergic neurotoxicity of MPTP and inflammogen lipopolysaccharide: relevance to the etiology of Parkinson's disease.. Faseb J.

[39] Rutten BP, Van der Kolk NM, Schafer S, van Zandvoort MA, Bayer TA (2005). Age-related loss of synaptophysin immunoreactive presynaptic boutons within the hippocampus of APP751SL, PS1M146L, and APP751SL/PS1M146L transgenic mice.. Am J Pathol.

[40] Wedenig M, Hutter-Paier B, Crailsheim K, Marksteiner M, Windisch M (2004). Serious behavioral and histological alteration in a transgenic mouse model overexpressing double-mutant human APP.. Neurobiology of Aging Volume.

[41] Deshusses JM, Burgess JA, Scherl A, Wenger Y, Walter N (2003). Exploitation of specific properties of trifluoroethanol for extraction and separation of membrane proteins.. Proteomics.

[42] Valtier D, Dement WC, Mignot E (1992). Monoaminergic uptake in synaptosomes prepared from frozen brain tissue samples of normal and narcoleptic canines.. Brain Res.

[43] Qin L, Wu X, Block ML, Liu Y, Breese GR (2007). Systemic LPS causes chronic neuroinflammation and progressive neurodegeneration.. Glia.

[44] Paxinos GF, J KB (1997). The Mouse Brain in StereotaxicCoordinates..

[45] Block ML, Wu X, Pei Z, Li G, Wang T (2004). Nanometer size diesel exhaust particles are selectively toxic to dopaminergic neurons: the role of microglia, phagocytosis, and NADPH oxidase.. FASEB J.

[46] Cheepsunthorn P, Radov L, Menzies S, Reid J, Connor JR (2001). Characterization of a novel brain-derived microglial cell line isolated from neonatal rat brain.. Glia.

[47] Floden AM, Combs CK (2006). Beta-amyloid stimulates murine postnatal and adult microglia cultures in a unique manner.. J Neurosci.

[48] Block ML, Li G, Qin L, Wu X, Pei Z (2006). Potent regulation of microglia-derived oxidative stress and dopaminergic neuron survival: substance P vs. dynorphin.. FASEB J.

[49] Peskin AV, Winterbourn CC (2000). A microtiter plate assay for superoxide dismutase using a water-soluble tetrazolium salt (WST-1).. Clin Chim Acta.

[50] Liu B, Hong JS (2003). Primary rat mesencephalic neuron-glia, neuron-enriched, microglia-enriched, and astroglia-enriched cultures.. Methods Mol Med.

[51] Tan AS, Berridge MV (2000). Superoxide produced by activated neutrophils efficiently reduces the tetrazolium salt, WST-1 to produce a soluble formazan: a simple colorimetric assay for measuring respiratory burst activation and for screening anti-inflammatory agents.. J Immunol Methods.

[52] Werner E (2003). Determination of cellular H2O2 production..

[53] Pei Z, Cheung RT (2003). Melatonin protects SHSY5Y neuronal cells but not cultured astrocytes from ischemia due to oxygen and glucose deprivation.. J Pineal Res.

[54] Qian L, Block ML, Wei SJ, Lin CF, Reece J (2006). Interleukin-10 protects lipopolysaccharide-induced neurotoxicity in primary midbrain cultures by inhibiting the function of NADPH oxidase.. J Pharmacol Exp Ther.

[55] Bradley MA, Markesbery WR, Lovell MA (2010). Increased levels of 4-hydroxynonenal and acrolein in the brain in preclinical Alzheimer disease.. Free Radic Biol Med.

[56] Sultana R, Butterfield DA (2009). Proteomics identification of carbonylated and HNE-bound brain proteins in Alzheimer's disease.. Methods Mol Biol.

[57] Sayre LM, Zelasko DA, Harris PL, Perry G, Salomon RG (1997). 4-Hydroxynonenal-derived advanced lipid peroxidation end products are increased in Alzheimer's disease.. J Neurochem.

[58] Hickman SE, Allison EK, El Khoury J (2008). Microglial dysfunction and defective beta-amyloid clearance pathways in aging Alzheimer's disease mice.. J Neurosci.

[59] Lee CY, Landreth GE The role of microglia in amyloid clearance from the AD brain.. J Neural Transm.

[60] Rockenstein E, Mallory M, Mante M, Sisk A, Masliaha E (2001). Early formation of mature amyloid-beta protein deposits in a mutant APP transgenic model depends on levels of Abeta(1-42).. J Neurosci Res.

[61] Hutter-Paier B, Huttunen HJ, Puglielli L, Eckman CB, Kim DY (2004). The ACAT inhibitor CP-113,818 markedly reduces amyloid pathology in a mouse model of Alzheimer's disease.. Neuron.

[62] Willis M, Hutter-Paier B, Wietzorrek G, Windisch M, Humpel C (2007). Localization and expression of substance P in transgenic mice overexpressing human APP751 with the London (V717I) and Swedish (K670M/N671L) mutations.. Brain Res.

[63] McGeer PL, Rogers J, McGeer EG (2006). Inflammation, anti-inflammatory agents and Alzheimer disease: the last 12 years.. J Alzheimers Dis.

[64] Jantaratnotai N, Schwab C, Ryu JK, McGeer PL, McLarnon JG (2010). Converging Perturbed Microvasculature and Microglial Clusters Characterize Alzheimer Disease Brain.. Curr Alzheimer Res.

[65] McGeer EG, McGeer PL (2010). Neuroinflammation in Alzheimer's disease and mild cognitive impairment: a field in its infancy.. J Alzheimers Dis.

[66] Hui-guo L, Kui L, Yan-ning Z, Yong-jian X (2010). Apocynin attenuate spatial learning deficits and oxidative responses to intermittent hypoxia.. Sleep Med.

