# Supplementary material for: Chronic Apocynin Treatment Attenuates Beta Amyloid Plaque Size and Microglial Number in hAPP(751)SL Mice
Source: PLoS One. 2011 May 31;6(5):e20153. doi: 10.1371/journal.pone.0020153 (PMC3105011; doi:10.1371/journal.pone.0020153)
Supplement: Table S1 — Microglial number in the hippocampus of hAPP(751)SL mice. The number of microglia was measured in hAPP(751)SL mice (Vehicle, DM 15 mg/kg, DM 7.5 mg/kg, and Apocynin 10 mg/kg) following 4 months of treatment. While the total number of microglia decreased significantly in the cortex (Figure 2), the number in the hippocampus showed only a trend toward a decrease in the numbers, as seen by staining with anti-CD11b antibody (statistical significance was tested with 1-way ANOVA with Bonferroni post-hoc test). (DOC) [file pone.0020153.s001.doc]

**Table S1. Microglial number in the hippocampus of hAPP(751)_SL_ mice.**

| **Group** | **Number of Cells per mm^2^** | **SEM** | **p-value vs. Vehicle*** |
| --- | --- | --- | --- |
| Vehicle | 15.13 | 2.08 | N/A |
| DM 15mg/kg | 17.78 | 2.41 | N/A |
| DM 7.5mg/kg | 13 | 1.98 | N/A |
| Apocynin 10mg/kg | 13.94 | 2.6 | N/A |
| **ANOVA P= 0.245** |  |  |  |

The number of microglia was measured in hAPP(751)_SL_ mice (Vehicle, DM 15mg/kg, DM 7.5mg/kg, and Apocynin 10mg/kg) following 4 months of treatment. While the total number of microglia decreased significantly in the cortex (Figure 2), the number in the hippocampus showed only a trend toward a decrease in the numbers, as seen by staining with anti-CD11b antibody (statistical significance was tested with 1-way ANOVA with Bonferroni post-hoc test).
