# Supplementary material for: Chronic Apocynin Treatment Attenuates Beta Amyloid Plaque Size and Microglial Number in hAPP(751)SL Mice
Source: PLoS One. 2011 May 31;6(5):e20153. doi: 10.1371/journal.pone.0020153 (PMC3105011; doi:10.1371/journal.pone.0020153)
Supplement: Table S2 — Behavioral measures in hAPP(751)SL mice. hAPP(751)SL mice (Vehicle, DM 15 mg/kg, DM 7.5 mg/kg, and Apocynin 10 mg/kg) were behaviorally tested using the Morris Water Maze. Measures of time learning (escape latency), length learning (length of swimming path), abidance in the target quadrant (% of total time), and number of target crossings were made for each animal, and values were compared to vehicle treated animals and tested for statistical significance (1-way ANOVA with Bonferroni post-hoc test, where applicable). No statistically significant changes were observed for any of the measurements, although trends toward improvement were seen in some tests. * p-value vs. vehicle is displayed when there was a trend (p<0.1) in ANOVA data.” Where trends were seen (ANOVA p<0.1) the p-value versus control is shown. (DOC) [file pone.0020153.s002.doc]

**Table S2. Behavioral measures in hAPP(751)_SL_ mice.**

|  |  | **Day 1** |  |  |
| --- | --- | --- | --- | --- |
| **Time Learning** | **Group** | **Average Escape Latency (sec)** | **SEM** | **p-value vs. Vehicle*** |
|  | Vehicle | 58.2 | 1.23 | N/A |
|  | DM 15mg/kg | 58.47 | 1.24 | N/A |
|  | DM 7.5mg/kg | 56.9 | 2.08 | N/A |
|  | Apocynin 10mg/kg | 55.15 | 3.28 | N/A |
|  | **ANOVA P=0.985** |  |  |  |
|  |  |  |  |  |
|  |  | **Day 2** |  |  |
|  | **Group** | **Average Escape Latency (sec)** | **SEM** | **p-value vs. Vehicle*** |
|  | Vehicle | 44.46 | 3.65 | N/A |
|  | DM 15mg/kg | 51.62 | 2.93 | N/A |
|  | DM 7.5mg/kg | 45.29 | 3.69 | N/A |
|  | Apocynin 10mg/kg | 38.89 | 5.14 | N/A |
|  | **ANOVA P=0.170** |  |  |  |
|  |  |  |  |  |
|  |  | **Day 3** |  |  |
|  | **Group** | **Average Escape Latency (sec)** | **SEM** | **p-value vs. Vehicle*** |
|  | Vehicle | 44.85 | 2.66 | N/A |
|  | DM 15mg/kg | 39.6 | 4.84 | N/A |
|  | DM 7.5mg/kg | 37.18 | 5.19 | N/A |
|  | Apocynin 10mg/kg | 42.62 | 4.8 | N/A |
|  | **ANOVA P=0.701** |  |  |  |
|  |  |  |  |  |
|  |  | **Day 4** |  |  |
|  | **Group** | **Average Escape Latency (sec)** | **SEM** | **p-value vs. Vehicle*** |
|  | Vehicle | 32.54 | 3.94 | N/A |
|  | DM 15mg/kg | 36.21 | 5.04 | N/A |
|  | DM 7.5mg/kg | 36.97 | 5.08 | N/A |
|  | Apocynin 10mg/kg | 32.51 | 4.18 | N/A |
|  | **ANOVA P=0.848** |  |  |  |

|  |  | **Day 1** |  |  |
| --- | --- | --- | --- | --- |
| **Length Learning** | **Group** | **Length of Swimming Path (m)** | **SEM** | **p-value vs. Vehicle*** |
|  | Vehicle | 6.25 | 0.39 | N/A |
|  | DM 15mg/kg | 6.82 | 0.57 | N/A |
|  | DM 7.5mg/kg | 6.57 | 0.57 | N/A |
|  | Apocynin 10mg/kg | 5.79 | 0.54 | N/A |
|  | **ANOVA P=0.537** |  |  |  |

|  |  |  |  |  |
| --- | --- | --- | --- | --- |
|  |  | **Day 2** |  |  |
|  | **Group** | **Length of Swimming Path (m)** | **SEM** | **p-value vs. Vehicle*** |
|  | Vehicle | 5.71 | 0.5 | N/A |
|  | DM 15mg/kg | 5.85 | 0.53 | 0.853 |
|  | DM 7.5mg/kg | 5.31 | 0.69 | 0.634 |
|  | Apocynin 10mg/kg | 3.98 | 0.52 | 0.025 |
|  | **ANOVA P=0.084** |  |  |  |
|  |  |  |  |  |
|  |  | **Day 3** |  |  |
|  | **Group** | **Length of Swimming Path (m)** | **SEM** | **p-value vs. Vehicle*** |
|  | Vehicle | 5.45 | 0.36 | N/A |
|  | DM 15mg/kg | 4.25 | 0.57 | N/A |
|  | DM 7.5mg/kg | 4.65 | 0.81 | N/A |
|  | Apocynin 10mg/kg | 4.76 | 0.57 | N/A |
|  | **ANOVA P=0.518** |  |  |  |
|  |  |  |  |  |
|  |  | **Day 4** |  |  |
|  | **Group** | **Length of Swimming Path (m)** | **SEM** | **p-value vs. Vehicle*** |
|  | Vehicle | 3.68 | 0.5 | N/A |
|  | DM 15mg/kg | 3.68 | 0.45 | N/A |
|  | DM 7.5mg/kg | 4.25 | 0.58 | N/A |
|  | Apocynin 10mg/kg | 3.46 | 0.59 | N/A |
|  | **ANOVA P=0.759** |  |  |  |

| **Abidance** | **Group** | **% of Time in Target Quadrant** | **SEM** | **p-value vs. Vehicle*** |
| --- | --- | --- | --- | --- |
|  | Vehicle | 32.31 | 2.05 | N/A |
|  | DM 15mg/kg | 25.83 | 5.07 | N/A |
|  | DM 7.5mg/kg | 34.55 | 4.79 | N/A |
|  | Apocynin 10mg/kg | 37.08 | 5.51 | N/A |
|  | **ANOVA P=0.338** |  |  |  |

|  |  |  |  |  |
| --- | --- | --- | --- | --- |
| **Target Crossings** | **Group** | **Number of Target Crossings** | **SEM** | **p-value vs. Vehicle*** |
|  | Vehicle | 2.85 | 0.37 | N/A |
|  | DM 15mg/kg | 2.25 | 0.45 | N/A |
|  | DM 7.5mg/kg | 3.18 | 0.58 | N/A |
|  | Apocynin 10mg/kg | 3.42 | 0.74 | N/A |
|  | **ANOVA P=0.475** |  |  |  |
|  |  |  |  |  |
|  | * p-value vs. Vehicle (t-test) is shown when a trend is seen in the data (ANOVA p<0.1); | | | |
|  | N/A reported when no trend is seen | |  |  |

hAPP(751)_SL_ mice (Vehicle, DM 15mg/kg, DM 7.5mg/kg, and Apocynin 10mg/kg) were behaviorally tested using the Morris Water Maze. Measures of time learning (escape latency), length learning (length of swimming path), abidance in the target quadrant (% of total time), and number of target crossings were made for each animal, and values were compared to vehicle treated animals and tested for statistical significance (1-way ANOVA with Bonferroni post-hoc test, where applicable). No statistically significant changes were observed for any of the measurements, although trends toward improvement were seen in some tests. * p-value vs. vehicle is displayed when there was a trend (p<0.1) in ANOVA data.” Where trends were seen (ANOVA p<0.1) the p-value versus control is shown.
