# Supplementary material for: Chronic Apocynin Treatment Attenuates Beta Amyloid Plaque Size and Microglial Number in hAPP(751)SL Mice
Source: PLoS One. 2011 May 31;6(5):e20153. doi: 10.1371/journal.pone.0020153 (PMC3105011; doi:10.1371/journal.pone.0020153)
Supplement: Table S3 — The effect of apocynin on levels of nitro-tyrosine in hAPP(751)SL mice. Following 4 months of treatment with Vehicle, DM 15 mg/kg, DM 7.5 mg/kg, or Apocynin 10 mg/kg, total protein was isolated from the brains of hAPP mice and the levels of nitrotyrosine were determined by ELISA (Millipore). No significant changes were observed in the levels of nitro-tyrosine between vehicle and treated mice. However, it is of note that the levels are very low, even in the vehicle treated hAPP transgenic mice (statistical significance was tested with 1-way ANOVA). (DOC) [file pone.0020153.s003.doc]

**Table S3. The effect of apocynin on levels of nitro-tyrosine in hAPP(751)_SL_** mice.

| **Group** | **Nitro-tyrosine (µg/mL)** | **SEM** | **p-value vs. Vehicle*** |
| --- | --- | --- | --- |
| Vehicle | 1.32 | 0.67 | N/A |
| DM 15mg/kg | 4.59 | 3.06 | N/A |
| DM 7.5mg/kg | 6.67 | 1.88 | N/A |
| Apocynin 10mg/kg | 2.12 | 0.78 | N/A |
| **ANOVA P=0.165** |  |  |  |

Following 4 months of treatment with Vehicle, DM 15mg/kg, DM 7.5mg/kg, or Apocynin 10mg/kg, total protein was isolated from the brains of hAPP mice and the levels of nitrotyrosine were determined by ELISA (Millipore). No significant changes were observed in the levels of nitro-tyrosine between vehicle and treated mice. However, it is of note that the levels are very low, even in the vehicle treated hAPP transgenic mice (statistical significance was tested with 1-way ANOVA).
